# USP13: Multiple Functions and Target Inhibition

**DOI:** 10.3389/fcell.2022.875124

**Published:** 2022-04-04

**Authors:** Xiaolong Li, Ge Yang, Wenyao Zhang, Biying Qin, Zifan Ye, Huijing Shi, Xinmeng Zhao, Yihang Chen, Bowei Song, Ziqing Mei, Qi Zhao, Feng Wang

**Affiliations:** ^1^ Key Laboratory of Molecular Medicine and Biotherapy, Department of Biology, School of Life Science, Beijing Institute of Technology, Beijing, China; ^2^ School of Chemistry and Biological Engineering, University of Science and Technology Beijing, Beijing, China; ^3^ University of Macau, China

**Keywords:** deubiquitination, ubiquitin-specific protease 13, structure, disease, inhibitor

## Abstract

As a deubiquitination (DUB) enzyme, ubiquitin-specific protease 13 (USP13) is involved in a myriad of cellular processes, such as mitochondrial energy metabolism, autophagy, DNA damage response, and endoplasmic reticulum-associated degradation (ERAD), by regulating the deubiquitination of diverse key substrate proteins. Thus, dysregulation of USP13 can give rise to the occurrence and development of plenty of diseases, in particular malignant tumors. Given its implications in the stabilization of disease-related proteins and oncology targets, considerable efforts have been committed to the discovery of inhibitors targeting USP13. Here, we summarize an overview of the recent advances of the structure, function of USP13, and its relations to diseases, as well as discovery and development of inhibitors, aiming to provide the theoretical basis for investigation of the molecular mechanism of USP13 action and further development of more potent druggable inhibitors.

## Introduction

**FIGURE 1:**
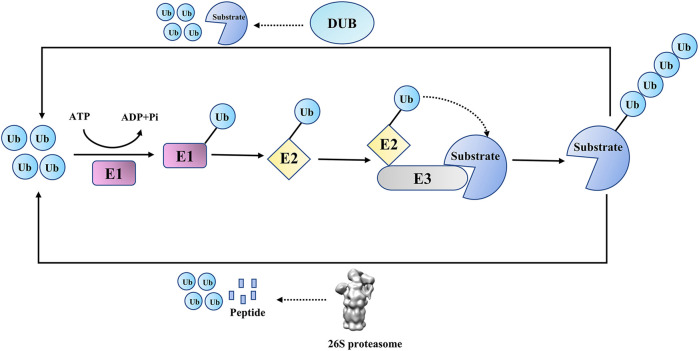
Ubiquitin proteasome pathway and deubiquitination.

Ubiquitination, as a crucial post-translational modification in eukaryotic cells, is involved in various cellular activities, including DNA damage repair (DDR), cell signal transduction, cell cycle regulation, and innate immune signaling pathways (Harrigan et al., 2018; Ciechanover, 2003; Ravid and Hochstrasser, 2008; Luan et al., 2016). In the process of ubiquitination, the ubiquitin (Ub) molecule is covalently attached to substrate proteins (or ubiquitin itself) through isopeptide bonds or peptide bonds by the E1-E2-E3 ligase cascade (or LUBAC complex) (Ciechanover, 2003; Dittmar and Winklhofer, 2019). Like other post-translational modifications, ubiquitination is reversible, and its reverse process, deubiquitination, is catalyzed by DUBs(Dandrea and Pellman, 1998) (Figure 1). DUBs can remove ubiquitins from substrate proteins (or poly-ubiquitin chains), edit ubiquitin chains and process ubiquitin precursors (Komander et al., 2009). These two processes coordinate to accurately maintain the proteostasis and ubiquitin balance in quantity.

To date, seven structurally distinct DUB families have been described, including ubiquitin-specific proteases (USPs), ovarian tumor proteases (OTUs), ubiquitin C-terminal hydrolase (UCHs), Machado–Josephin domain-containing proteases (MJDs), motifs interacting with the ubiquitin-containing novel DUB family (MINDYs), JAB1, MPN, MOV34 family (JAMMs), and zinc finger containing Ub peptidase 1 (ZUP1) (Kwasna et al., 2018; Cho et al., 2020; Wang and Wang, 2021). JAMMs are zinc metallopeptidases, while the other six DUB families are cysteine peptidases. The USPs family has the largest number of members with diverse functions, providing the potential for developing drugs with more specific effects (Sippl et al., 2011; Yuan et al., 2018; Cruz et al., 2021).

USP13, belonging to the USPs family, is known to be extensively engaged in diverse cellular processes, such as mitochondrial energy metabolism, autophagy, DNA damage response, ERAD and other processes, by deubiquitinating substrates α-ketoglutarate dehydrogenase (OGDH) (Han et al., 2016), ATP citrate lyase (ACLY) (Han et al., 2016), vacuolar protein sorting 34 (VPS34) (Xie et al., 2020), topoisomerase IIβ binding protein 1 (TopBP1) (Kim et al., 2021), receptor-associated protein 80 (RAP80) (Li et al., 2017), and ubiquitin like 4A (UBL4A) (Liu et al., 2014). Ample findings prove that USP13 may also promote the initiation or progression of various tumors. For example, the stabilization of microphthalmia-associated transcription factor (MITF) by USP13 was found to be associated with proliferation of melanoma cells (Zhao et al., 2011); USP13 is abnormally overexpressed in ovarian cancer (OVCA) and drives OVCA metabolism to accelerate cell proliferation through deubiquitinating ACLY and OGDH (Han et al., 2016); in glioblastoma, USP13 promotes the proliferation of glioma stem cells (GSCs) by antagonizing E3 ubiquitin ligase F-box and leucine-rich repeat protein 14 (FBXL14), which inhibits the ubiquitination and degradation of pro-oncogene c-Myc (Fang et al., 2017); in non-small-cell lung cancer (NSCLC), downregulation of USP13 impedes the growth of NSCLC model cells A549 and H226 via suppressing AKT/MAPK signaling pathway (Wu et al., 2019); in colorectal tumor cells, USP13 has been identified as a microRNA-135b24 target that promotes colorectal tumor cell proliferation and glycolysis (Zhang et al., 2013).

Incompatible with the above findings, the recombinant expression of USP13 exhibits only weak deubiquitination enzyme activity *in vitro* (Liu et al., 2011; Zhang et al., 2011). To decipher its activation mechanism for interpretating the paradoxical phenomena, determining USP13 structure has attracted considerable interest over the past few years. Albeit the structures of full-length USP13, as well as its catalytic structural domain have not been obtained, structures of several functional domains are determined (Liu et al., 2011; Zhang et al., 2011; Liu et al., 2014; Han et al., 2016). Given the implications of USP13 in tumorigenesis, seeking compounds that modulate the USP13 emerges an active area of research and achieves impressive progress, with multiple selective compounds being identified successively by both research institutions and pharmaceutical companies (Liu et al., 2011; Liu et al., 2021a).

In this review, we discuss recent advances in our understanding of the physiological roles, structure, and USP13-related diseases. In addition, the appealing stories regarding a range of representative small-molecule inhibitors are listed to help track their evolution.

### Structure and Activation Mechanism of USP13

**FIGURE 2:**
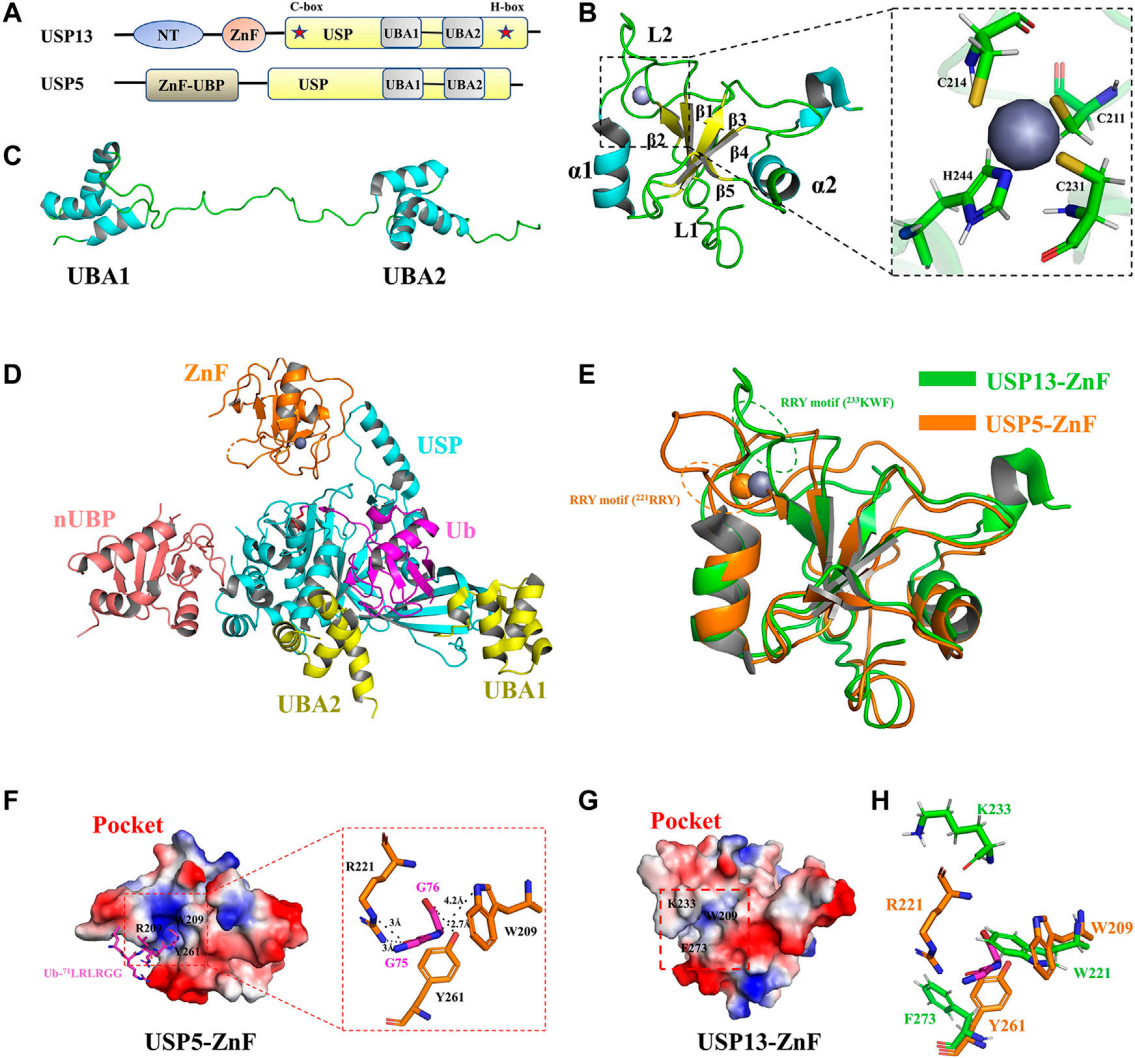
Structure of USP13 and comparison with USP5-ZnF. **(A)** Domain structure of USP13 and USP5. **(B)** Structure of USP13-ZnF (PDB 2L80), in which the α helix is blue, the β sheet is yellow, the loop is green, and the zinc ion is gray. The close-up view shows that the zinc nucleus coordinates with the peptide chain in C3H mode. **(C)** Structure of USP13-UBA (PDB 2LBC), in which the α helix is blue and the loop is green. **(D)** Structure of ubiquitin-USP5 complex (3IHP). nUBP is pink, ZnF is orange, the USP catalytic domain is blue and UBA12 is yellow. Ubiquitin is purple. **(E)** Comparison of the structure between USP13-ZnF and USP5-ZnF (PDB 2G43), USP13-ZnF and USP5-ZnF are green and orange, respectively. **(F)** Electrostatic surface of the Ubiquitin-USP5-ZnF complex (PDB 2G45). Ubiquitin glycine motif (^71^LRLRGG, purple) is inserted into the ubiquitin binding pocket of USP5-ZNF, and it can be seen from the close-up view that ubiquitin G75 and G76 form hydrogen bonds interact with W209, R221, and Y261 on ZnF. **(H)** Electrostatic surface of the USP13-ZnF. **(G)** The residues of the combined Ub-G75 and Ub-G76 on USP5 (orange) are displayed in sticks, and the corresponding residues in USP13 (green) are displayed in sticks.

The *usp13* gene is located on human chromosome 3q26.2–q26.3, which encodes USP13, also known as isopeptidase T-3 (Zhang et al., 2011; Timms et al., 1998). USP13 was first identified by Timms *et al.* and consisted of 863 amino acids (Timms et al., 1998). USP13 shares approximately 80% sequence similarity with USP5(Zhang et al., 2011). They have the same domain architecture, including the N-terminal domain, Zinc finger (ZnF) domain (amino acids 209–281), and USP catalytic domain (amino acids 336–861), between the C-box and H-box (including a two-UBA insertion) (Zhang et al., 2011; Ning et al., 2020) (Figures 2A,D). The N-terminal residues of USP13 might be essential for physical interaction with other proteins, which could be exemplified by interaction of the N-terminus of USP13 with myeloid cell leukemia sequence 1 (MCL1), a core member of the anti-apoptotic B cell lymphoma 2 (BCL-2) family of proteins (Zhang et al., 2018). As the ZnF domain is generally considered to be a ubiquitin binding site, USP5-ZnF recognizes the C-terminal glycine motif of free Ub chains and activates deubiquitination, while USP13-ZnF domain is unable to bind Ub, although the sequences of the ZnF domains from these two USPs are homologous (Zhang et al., 2011; Reyes-Turcu et al., 2006) (Figure 2B, Supplementary Figure S1). The USP13 catalytic domain contains a conserved C-box and H-box including a two-UBA insertion (Timms et al., 1998; Zhang et al., 2011). Although the experimental results showed that USP13-UBA could bind ubiquitin, USP13 still exhibited only weak deubiquitination enzyme activity, which is incompatible with the findings that USP13 can deubiquitinate various important substrates implicated in disease and tumor development (Liu et al., 2011; Zhang et al., 2011). It is assumed that USP13 is constitutively in a state of self-inhibition, whereas it can be activated when it is modified or interacts with other proteins. However, the structure of USP13 catalytic domain has not been available until now, at large impeding interpretation of its active mechanism at the atomic level.

Fortunately, the USP13-ZnF domain and the tandem UBA domain have been obtained using NMR (Zhang et al., 2011). In 2011, Hu et al. reported the solution structure of USP13-ZnF (PDB 2L80). As shown in Figure 2B, USP13-ZnF contains only one zinc nucleus that coordinates with the peptide chain in C3H mode. The USP13-ZnF domain consists of five anti-parallel β sheets and two α helices located on both sides with a flexible loop connecting them (named Loop 2, L2). In contrast with USP13-ZnF domain, USP5-ZnF binds Ub with a comparatively high affinity, echoing the distinct structures from two ZnF domains (Reyes-Turcu et al., 2006). Structural comparison revealed no significant difference (RMSD = 1.47) between USP13-ZnF and USP5-ZnF, but there were some slight differences between the two USPs (Figure 2E). Firstly, USP13-ZnF and USP5-ZnF bind Ub with distinct pockets: the pocket USP13-ZnF appears shallower and harbors few positive charges than that of USP5-ZnF, which is not conducive to the binding of Ub glycine (Figures 2F,G); the L2 regions in the two ZnF domains are moderately different, and Arg-Arg-Try motif (RRY motif) in L2 regions are likely associated with ubiquitin binding (Figure 2E). In addition, W209, R221 and Y261 on USP5 form hydrogen bonds with Ub-75G and Ub-76G, which are key residues for Ub binding (Figure 2F). However, it is speculated from the structural alignment (Figure 2H, Supplementary Figure S1) that W221, K233, and F273 corresponding to USP13 are not completely conservative, especially since K233 is offset from the pocket (K233 at L2 region) (Zhang et al., 2011). In conclusion, USP13-ZnF failed to bind Ub for two reasons: one is distribution of the binding pocket charge; and the other is change of conserved ubiquitin-binding residues.

Both USP13 and USP5 contain tandem UBA domains inserted between the C-box and H-box, and UBA contains the very conserved Ub binding motif Met-Gly-Phe (MGF) (Timms et al., 1998; Raasi et al., 2005). In 2011, Hu et al. reported the solution structure of USP13-UBA (PDB 2LBC). Structural analysis demonstrated that UBA consists of three α-helices, and there was no direct interaction between the two UBAs, which were linked by a long loop (Figure 2C). Albeit no ubiquitin-bound UBA structure is resolved, sequence alignment revealed that USP13-UBA contains an MGF motif that presumably can bind Ub (Supplementary Figure S1), consistent with results from pull-down and ITC experiments (Zhang et al., 2011). In addition, NMR titration data reflected that M664, F666, M739, and F741 might be the key residues responsible for the binding of USP13-UBA to Ub (Zhang et al., 2011). Except for binding Ub, USP13-UBA2 has also been reported to be required for binding other proteins, such as the E3 ubiquitin ligase glycoprotein 78 (gp78) (Liu et al., 2014).

In spite of the high similarity between USP13 and USP5 both in sequence and domains structures, USP13 recombinant protein exhibits weak deubiquitination activity *in vitro*, dramatically different from its homolog USP5 with high activity both *in vivo* and *in vitro*. In the Ubiquitin-7-amido-4-methylcoumarin (Ub-AMC) hydrolysis experiment, USP5 showed high deubiquitination activity at 1.5 nM, while USP13 only has displayed extremely weak activity until protein concentration increased to 500 nM(Zhang et al., 2011). In the ubiquitin chain hydrolysis experiment, USP5 can hydrolyze into anchored ubiquitin chains one by one from the near end until all ubiquitin chains are cleaved into single ubiquitin chains, and all polyubiquitin chains, Lys-48 and Lys-63 linear ubiquitin chains, can be recognized and cleaved by USP5 (Amerik Ayu et al., 1997; Reyes-Turcu et al., 2006; Reyes-Turcu et al., 2008). However, the experiment proved that USP13 has no hydrolytic activity to Lys-48 and Lys-63 chain diubiquitin but can slowly hydrolyze Lys-63 chain tetraubiquitin to triubiquitin and monoubiquitin (Zhang et al., 2011). Overall, the USP13-ZnF domain cannot bind to Ub to activate USP13, whereas USP13-UBA can bind, which may partially explain the reason why USP13 displays only weak basal deubiquitination enzyme activity: the binding sites of USP13 to Ub are less than that other USP members, thus providing weaker binding affinity and consequent cleavage activity towards ubiquitin chains; There possibly exists constitutive self-inhibition for full-length USP13 supported by the interaction of UBA with ZnF domain, which is hypothesized to be released by recruitment of other proteins or modification, such as phosphorylation. However, no relevant research progress is reported to verify this hypothesis at present.

## Cellular Function of USP13

### USP13 in Energy Metabolism

**FIGURE 3:**
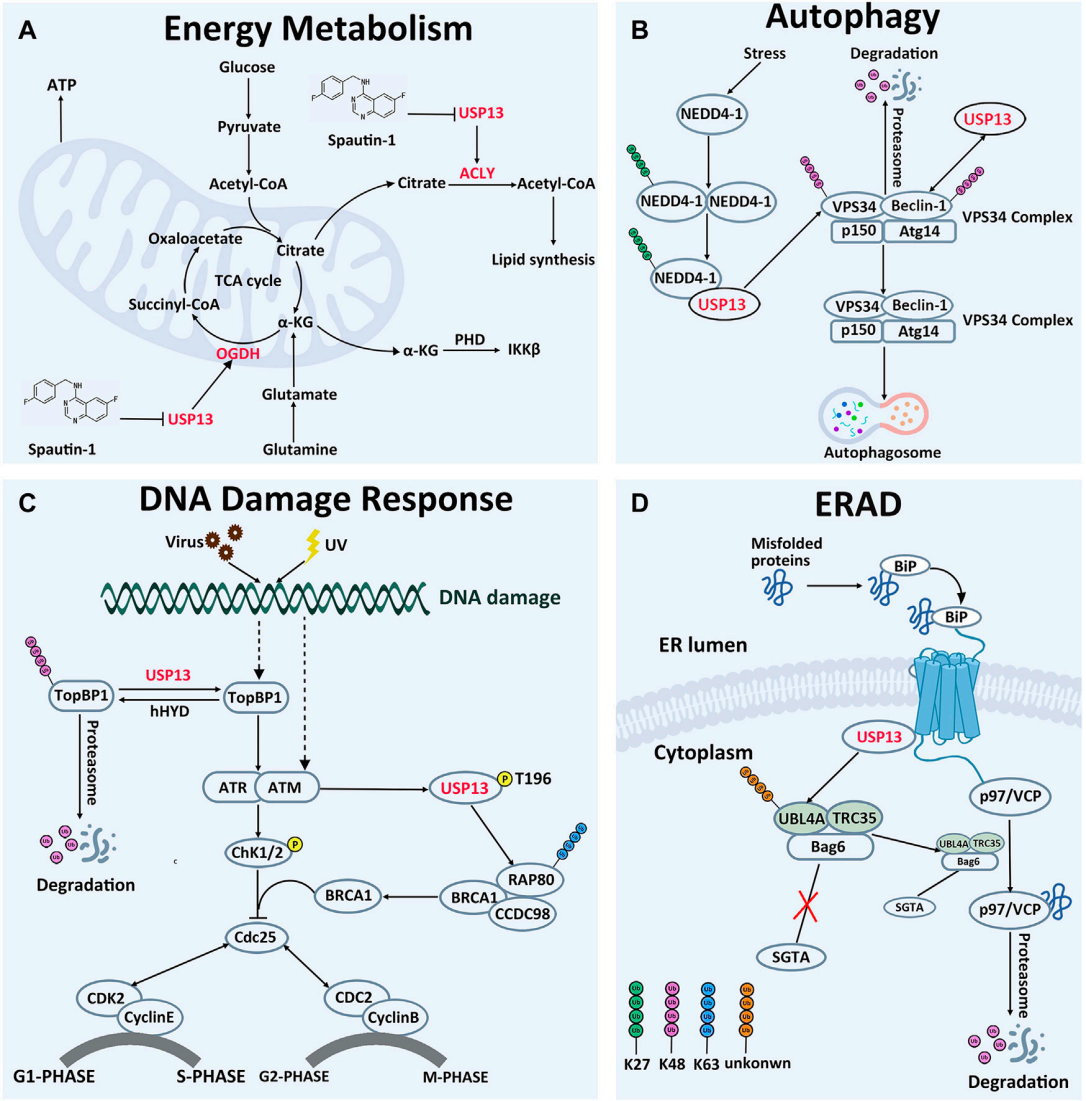
Cellular Function of USP13. **(A)** In energy metabolism, USP13 promotes cell energy metabolism by removing OGDH and ACLY degradation signals. **(B)** In autophagy, USP13 is recruited by auto-ubiquitinated NEDD4-1 to remove VPS34 subunit degradation signal. On the other hand, USP13 deubiquitinated Beclin-1 and removed the degradation signal of Beclin-1. Consequently, the VPS34 complex initiates autophagosome formation. K27-linked Ub chains are green, K48-linked Ub chains are pink. **(C)** In DNA damage reaction, USP13 antagonizes E3 ubiquitin ligase hHYD to remove the TopBP1 degradation signal, then TopBP1 activates the ATR signaling pathway and ultimately activates the G1-S phase checkpoint, making cells remain in the G1 phase. In addition, phosphorylated USP13 by ATM is recruited to DNA damage sites to cleave the other Ub chains to facilitate K63-linked ubiquitin chains, which promotes RAP80 localization and ultimately activates the G2-M phase checkpoint, making cells remain in the G2 phase. K48-linked Ub chains are pink. Phosphorylation is yellow. **(D)** In ERAD, hyper-ubiquitination of UBL4A can induce the cleavage and inactivation of Bag6, causing ERAD inhibition and inhibiting the interaction of UBL4A with SGTA directly. USP13 interacts physically with gp78 and Bag6, removing hyper-ubiquitination of UBL4A, controlling precisely the ERAD process. Unknown-linked Ub chains are orange.

The tricarboxylic acid cycle is the core pathway of energy metabolism and the hub of carbohydrate, lipid and amino acid metabolism, providing precursor molecules for the synthesis of various lipids, non-essential amino acids and nucleotides (Akram, 2014; Han et al., 2016; Salway, 2018). Studies have demonstrated that USP13 can regulate the cellular levels of two key proteins involved in mitochondrial energy metabolism (Han et al., 2016). In normal cells, glucose is converted to acetyl-CoA, entering into the tricarboxylic acid cycle and further generating citric acid (Gameiro and Bell, 2011; Gameiro et al., 2013). Part of citric acid is transported to the cytoplasm, where it is converted to acetyl-CoA by ACLY and eventually supplied for lipid synthesis (Hatzivassiliou et al., 2005). However, in most cases, glutamate intake in tumor cells would markedly ascend in order to provide more intermediates of the tricarboxylic acid cycle, maintaining lipid synthesis (Han et al., 2016). Glutamic acid is converted to α-ketoglutarate by glutaminase and glutamate dehydrogenase, entering into the tricarboxylic acid cycle. Subsequently, α-ketoglutarate is oxidized to succinic acid by OGDH to ensure smooth operation of the tricarboxylic acid cycle (Sun and Denko, 2014; Han et al., 2016) (Figure 3A).

Phosphatidylinositol-3-kinase (PI3K)/AKT is a well-recognized signaling pathway related to energy metabolism, and USP13 knockdown is demonstrated to enhance the sensitivity of OVCA cells to AKT inhibitors, implying the role of USP13 in PI3K/AKT-dependent energy metabolism (Hanrahan et al., 2012; Han et al., 2016; Xie et al., 2019). Consistently, ACLY and OGDH were identified as USP13 interacting proteins utilizing mass spectrometry (Han et al., 2016). Meanwhile, protein-binding assay and deubiquitylation experiments demonstrated that USP13 could interact with OGDH or ACLY through N-terminal domain or C-terminal domain, respectively, to remove K48-linked ubiquitin chains from ACLY and OGDH, stabilizing their intracellular protein levels. When WT-USP13 but not CA-USP13 (C345A-USP13 mutation, inactive mutation) was overexpressed, ACLY and OGDH protein concentrations in OVCA cells were upregulated, but mRNA levels were not significantly altered. Consistently, tissue microarray detected that USP13 knockdown could reduce the ACLY and OGDH protein levels in cells, reducing the synthesis of fatty acids from the source. As expected, USP13 knockout evidently inhibited tumor cell growth either in OVCA cells or xenograft tumor models in nonobese diabetic/severe combined immunodeficiency (NOD/SCID) mice (Han et al., 2016). In conclusion, inhibition of the deubiquitination activity of USP13 can impair the energy metabolism of tumor cells, hence providing novel insights into interventions of tumor cells targeting on energy metabolism pathway.

### USP13 in Autophagy

Autophagy is a process where cells self-degrade and recycles their intracellular organelles under stress or starvation. Disruption of the autophagy system may trigger the occurrence of tumors and autoimmune diseases (Levine and Kroemer, 2008; Glick et al., 2010; Levy et al., 2017). Data demonstrated that VPS34 PI3K activity and its protein partners play essential roles in harmonizing both autophagosome initiation and maturation (Liu et al., 2011). VPS34 and Beclin1 are the core components of the VPS34 complexes (Ohashi et al., 2019; Xie et al., 2020; Yang et al., 2021).

In 2011, Yuan *et al.* found that USP13 could interact with the C-terminal domain of Beclin-1 subunit in the VPS34 complex and deubiquitinate Beclin-1, thereby enhancing the stability of the VPS34 complex, which would contribute to the formation of autophagosomes (Liu et al., 2011). Deubiquitination experiments demonstrated that overexpression of USP13 could reduce the ubiquitination level of Beclin-1, and this effect could be counteracted by the USP13 inhibitor spautin-1. Interestingly, Beclin-1 knockout also reduced USP13 protein abundance in turn, indicating the two proteins are regulated reciprocally. In addition, a recent study pronounced another implication of USP13 in modulating autophagy through deubiquitinating VPS34 subunit (Xie et al., 2020). When autophagy occurs, neural precursor cells expressed developmentally downregulated 4-1 (NEDD4-1) would form oligomer and undergo K29-linked auto-ubiquitination at K1279. The auto-ubiquitinated NEDD4-1 can then interact with USP13 and act as a bridge connecting USP13 to VPS34, removing the K48-linked ubiquitin chain on VPS34. Furthermore, it was demonstrated that CA-USP13 is not able to cleave K48-linked ubiquitin chain, suggesting that USP13 deubiquitination activity is necessary to stabilize VPS34 (Figure 3B). Thus, modulating USP13 perhaps offers an effective target in the management of diseases brought by disfunction of the autophagy pathways. Consistently, treatment with spautin-1, a selective inhibitor of USP13, protected the brain from cerebral ischemia reperfusion injury through blocking autophagy activation (Liu et al., 2021b). In addition, upregulation of USP13 is proved to attenuate intervertebral disc degeneration (IVDD) through promoting autophagy (Dai et al., 2021).

### USP13 in DNA Damage Response

DNA replication is an important process of genetic information transmission, and imperfect replication processes lead to genomic instability, which is a critical cause of tumors (Downs et al., 2007; Anindya, 2020). Stimulus from external radiation, viral infection and other stimuli can trigger DNA damage in cells, initiating the DNA damage repair system to protect the DNA structure from destruction (Li et al., 2017). It is indicated that USP13 can exquisitely adjust several vital proteins involved in the DNA damage response through deubiquitinating them, in degradation-dependent and independent manner (Li et al., 2017; Kim et al., 2021). Here, we take its regulation of RAP80 and TopBP1 for instances to discuss (Figure 3C).

RAP80 exerts effects in myriad aspects of DNA damage repair, including cell cycle checkpoint activation and chromatin homologous recombination (Mailand et al., 2007; Silver and Livingston, 2012). It is observed that USP13 deficiency abrogates DNA damage-induced G2/M checkpoint and renders cells sensible to irradiation and treatment of cisplatin in a RAP80-dependent manner, underlying the implications of USP13 in DNA damage repair through modulating RAP80 (Li et al., 2017). The evidence establishes that the binding of RAP80 to K63-linked ubiquitin chain is essential for recruitment of itself and other proteins to DNA damage sites (Kim et al., 2007; Sobhian et al., 2007; Wang et al., 2007; Yan et al., 2007). However, there are approximately 15 sites on RAP80 prone to forming ubiquitin chains, and activation of multiple sites might sterically block modification of RAP80 by K63-linked ubiquitin chain. Following DNA damage, phosphorylated USP13 by ATM is recruited to DNA damage sites to cleave the ubiquitin chains from more than three sites of RAP80 (K75, K90, and K112), releasing their restriction on the K63-linked ubiquitin chain, improving the focus formation of the RAP80-BRCA1 complex, and eventually facilitating DDR. Notably, this function of USP13 depends on deubiquitination activity, since CA-USP13 cannot reduce RAP80 ubiquitination level (Li et al., 2017). Taken together, USP13 has an impact on ubiquitination of RAP80, instead of its protein degradation, to regulate its focus formation and DDR-related function. In addition, depletion of USP13 in OVCA cell line EFO-27 cells sensitized cells to the Poly (ADP-ribose) polymerase (PARP) inhibitor, Olaparib, and incubation with USP13 inhibitor Spautin-1 also conferred EFO-27 cells sensitive to Olaparib. Furthermore, following treatment of Spautin-1 in conjunction with Olaparib, effects on OVCA models are remarkably enhanced, indicating that USP13 may be applied to overcome the chemotherapy resistance of cancer cells (Li et al., 2017).

Alternatively, USP13 can also modulate the ubiquitination level of TopBP1, another key protein implicated in replication stress-related DNA-damage responses (Kim et al., 2021). Following DNA replication stress, TopBP1 is recruited near single-stranded DNA to activate the ATR, thereby regulating the G1-S phase checkpoint (Ma et al., 2020; Kim et al., 2021). In normal cells, TopBP1 is ubiquitinated by the E3 ubiquitin ligase human hyperplastic discs (hHYD) for degradation by the proteasome (Honda et al., 2002). Under DNA damage, the ubiquitination level of TopBP1 was pronouncedly reduced by USP13, accompanied by accumulation of TopBP1 in cells. Protein interaction experiments demonstrated that USP13 could co-immunoprecipitate with endogenous TopBP1, and *in vitro* deubiquitination enzyme experiments showed that WT-USP13 could deubiquitinate TopBP1, while CA-USP13 could not, highlighting the requirement for USP13 ubiquitination activity (Kim et al., 2021). The observations that ATR phosphorylation was reduced in USP13-deficient cells and can be restored by recombinant expression of TopBP1 established that USP13 can regulate DNA replication stress by controlling the degradation of the TopBP1. Importantly, TopBP1 is proved to be correlated with multiple cancers and exerts roles in chemotherapy resistance (Forma et al., 2012; Chowdhury et al., 2014; Lv et al., 2016; Liu et al., 2021c; Laine et al., 2021). Moreover, incubation with USP13 inhibitor spautin-1 reduces survival of OVCA cell lines after replication stress inducing agents, implying that the development of selective USP13 inhibitors is feasible for treatment of these patients of conventional cancer chemotherapy (Kim et al., 2021).

Conclusively, these data illustrate that USP13 can deubiquitinate key proteins engaged in DNA damage response to induce their dysfunction or degradation, fine-tuning the DNA damage repair system.

### USP13 in ERAD

To control protein quality in cells, proteins are strictly monitored in the endoplasmic reticulum. Those proteins that cannot be correctly folded will be degraded by the ERAD pathway, where the misfolded proteins should be moved by the process, named retrotranslocation, from the endoplasmic reticulum across the membrane to the cytosol for ubiquitination by ER-associated ubiquitin conjugating systems (Hirsch et al., 2009; Ye and Rape, 2009). As a recognition signal, the polyubiquitin chains on the substrates can enroll the p97/VCP ATPase and its cofactor Ufd1-Npl4, releasing substrates from the ER membrane into the cytosol (Ye et al., 2001; Flierman et al., 2003). It is reported that USP13 and gp78 are two enzymes with opposing activity, but manipulate in combination the ubiquitination of ER substrates, thus coordinately promoting ERAD (Liu et al., 2014) (Figure 3D). Gp78, as one of the well-described E3s in ERAD, plays a master regulator of retrotranslocation, via mediating ubiquitination of many ERAD substrates and interacting with ERAD machinery proteins, such as BCL-2-associated athanogene 6 (Bag6) multiprotein complex (Fang et al., 2001; Song et al., 2005; Jo et al., 2011; Wang et al., 2011; Chen et al., 2012). On the luminal side, a complex containing gp78 can recruit the misfolded proteins recognized by molecular chaperone proteins for ubiquitination and retrotranslocation (Brodsky and McCracken, 1999; Wu and Rapoport, 2018). Bag6 with chaperone “holdase” activity can improve the turnover of retrotranslocated polypeptides through holding them in a soluble state and facilitating the transfer of the substrate from the gp78 containing complex to the proteasome for degradation, owing to the weak interaction of Bag6 with the proteasome (Minami et al., 2010; Wang et al., 2011). UBL4A is one of two Bag6 partners, promotes association of Bag6 with a co-chaperone. Hyper-ubiquitination of UBL4A can induce the cleavage and inactivation of Bag6, causing ERAD inhibition (Chartron et al., 2012; Xu et al., 2012). Therefore, ubiquitination chains on UBL4A are essential for the ERAD pathway. It is reported that USP13 can form a specific interaction with the Bag6 complex via the Bag6 UBL domain, and further remove ubiquitin conjugates from UBL4, hyper-ubiquitination of UBL4 under USP13 knockdown conditions might inhibit the interaction of UBL4 with SGTA directly, and therefore disrupting this functional connection between Bag6 and SGTA (Liu et al., 2014; Chu et al., 2020). Data demonstrated that ubiquitin conjugates on Ubl4A from either USP13 deficient cells or USP13 knockdown cells accumulated more than that on UBL4 from control cells. Similarly, ubiquitinated UBL4A can be significantly reduced after treatment of with recombinant USP13, which can be blocked by the specific DUB inhibitor ubiquitin aldehyde (Ub-Al) (Liu et al., 2014). As a result, USP13 can inhibit the elimination of misfolded proteins by the ERAD pathway. Notably, the impact of deubiquitination enzyme USP13 on ERAD process needs the assistance of gp78, and in turn improves the ubiquitination specificity of gp78 substrates. USP13 interacts physically with gp78 and Bag6, fine-tuning the ubiquitin dynamics of UBL4A in the Bag6 complex. Gp78 adds ubiquitin chains into UBL4A, whereas USP13 antagonizes this activity to limit UBL4A ubiquitination. In conclusion, it appears that USP13 and gp78, these two antagonized enzymes against each other, corporately maintain the balance between ubiquitination and deubiquitination, controlling precisely the ERAD process.

It is noteworthy that USP13 also can act in ERAD downstream of retro-translocation through enhancing the solubility of retrotranslocated substrates (Liu et al., 2014; Chu et al., 2020). It is proved that USP13 knockdown has negative effects on the solubility of several ERAD substrates, including model ERAD-substrate TCRα. This phenomenon is postulated perhaps due to mutual influence between USP13 and Bag6 (Yu et al., 1997; Soetandyo et al., 2010). Lately, USP13 has been reported to deubiquitinate under stress, which is also the substrate of autocrine motility factor receptor (AMFR) E3 ligase, activating CASP3 followed by Bag6 cleavage (Mitchell et al., 2007; Benhar et al., 2008). Consequently, the produced N-terminal Bag6 is converted from an ERAD regulator to an autophagy modulator and apoptosis trigger.

### USP13 in Other Cellular Activities

In addition to these cell functions as described above, USP13 is also implicated in many other distinctive cell activities, albeit its regulatory mechanism remains not elucidated clearly. For instance, in non-small-cell lung cancer (NSCLC), downregulation of USP13 inhibits MAPK/AKT signaling (Han et al., 2016). In contrast, in breast cancer cells, silencing USP13 can facilitate AKT phosphorylation by downregulating PTEN level, accompanied by tumor cell proliferation and glycolysis (Zhang et al., 2013). As STING (also known as MITA), a deubiquitination substrate of USP13, is pivotal for host defense against viruses dependent on the NF-κB pathway and USP13 is supposed to be involved in the NF-κB signaling pathway and regulates innate immunity via deubiquitinating STING (Sun et al., 2017). Consistently, it has been reported that deletion of USP13 can activate the NF-κB signaling pathway in response to herpesvirus infection, increasing resistance to the virus (Sun et al., 2017). Moreover, phosphorylation of USP13 at Y708 by CDC-like kinase 3 (CLK3) can facilitate the interaction between USP13 and the proto-oncoprotein c-Myc, further suppressing tumorigenesis (Zhou et al., 2020). Overall, USP13 is capable of affecting various cellular processes, including protein localization or degradation through regulating the ubiquitination levels of multiple protein substrates, thereby the dysfunction of USP13 can relate to a wide variety of diseases, even the occurrence of tumors, which highlight the potency of USP13 as a therapeutic target.

### USP13 and Tumors

A growing number of studies have demonstrated that USP13 overexpression is closely related to tumor grade, tumor invasion, chemotherapy resistance and poor prognosis (Liu et al., 2014; Han et al., 2016; Fang et al., 2017; Li et al., 2017; Kim et al., 2021; Liu and Moussa, 2021).

The Cancer Genomics Atlas (TCGA) analysis detected significant overexpression of USP13 in OVCA cells. Immunohistochemical (IHC) assay exhibited USP13 expression levels in OVCA cells upregulated at least 3.7 times, compared with those in normal ovarian tissues (Han et al., 2016). In addition, clinical data showed that USP13 overexpression led to a short survival cycle and poor prognosis for OVCA patients and is closely related to tumor grade. Consistently, knockout or pharmacological inhibition of USP13 impeded tumor cell proliferation and enhanced sensitivity to chemotherapeutic agents in both cell lines and mouse models (Han et al., 2016; Zhang et al., 2018). Mechanistically, USP13 promoted the energy metabolism of tumor cells, and provided precursor substances for the synthesis of sugar, lipids and non-essential amino acids in cancer cells, through deubiquitinating and stabilizing ACLY and OGDH (Han et al., 2016). These are necessary for tumor cell proliferation and invasion. USP13, on the other hand, can deubiquitinate and stabilize MCL1, which is not sensitive to MCL-2 family inhibitors, and render tumor cells highly resistant to BH3-type chemotherapy drugs (Oltersdorf et al., 2005; Delbridge et al., 2016; Kotschy et al., 2016). Since the effect of USP13 on maintaining this resistance, inhibition of USP13 seems likely to be viewed as a practical way to overcome drug resistance in the therapy of OVCA.

The *c-Myc* gene encodes a proto-oncoprotein, a widely recognized transcription factor regulating approximately 10–15% of genes implicated in cell proliferation, differentiation, apoptosis and other processes (Friedman et al., 2017; Habib et al., 2020), and c-Myc mutations are often associated with tumors (Berns et al., 1997; Brodsky and McCracken, 1999; Hermeking et al., 2000; Morrish et al., 2003; Wilson et al., 2004). Recently, it has been reported that USP13 is co-overexpressed with c-Myc in many tumors, such as NSCLC (Wu et al., 2019), cholangiocarcinoma (CAA) (Zhou et al., 2020), GSCs (Fang et al., 2017), and hepatocellular carcinoma (HCC) (Huang et al., 2020). Consistently, knockdown or pharmacological inhibition of USP13 antagonized tumor cell growth. For example, in non-small-cell lung cancer, downregulation of USP13 suppresses ATK/MAPK signaling, reducing c-Myc protein levels and retards tumor growth both in tumor cells and nude mice (Wu et al., 2019). In cholangiocarcinoma, TGF-β signaling triggers the phosphorylation of CLK3, a serine/threonine kinase that directly phosphorylates USP13 at Y708 and facilitates USP13 interaction with c-Myc (Zhou et al., 2020); in GSCs, USP13 can enhance the stability through deubiquitinating c-Myc, activating purine synthesis mediated by c-Myc and inducing the tumorigenesis of GSCs (Fang et al., 2017); in hepatocellular carcinoma, knockdown of USP13 by shRNA can markedly downregulate c-Myc expression, resisting xenograft tumor growth of HCC (Huang et al., 2020). Hence, inhibition of USP13 might be beneficial for related cancer treatment.

Likewise, USP13 exerts an antitumor role in several types of cancers. For example, USP13 prevents tumor cell growth by deubiquitinating PTEN in breast cancer, OSCC and bladder cancer. It is assumed that overexpression of USP13 can block AKT signaling pathway, suppressing tumor cell proliferation, invasion, and glycolysis through up-regulating PTEN protein levels (Zhang et al., 2013; Xiang et al., 2015; Zhang et al., 2018; Man et al., 2019).

In addition, USP13 is also involved in the development of other diseases and tumors. In cell and animal models, USP13 participates in ubiquitination modifications of key targets in Parkinson’s disease, such as tau, α-synuclein and E3 ubiquitin ligase parkin (Liu et al., 2019a; Liu et al., 2019b). In melanoma, MITF is essential for cell proliferation and differentiation via regulating multiple genes transcription. USP13 has been identified as a deubiquitination enzyme of MITF to modulate the ubiquitination level of MITF, affecting the survival of melanoma cells (Zhao et al., 2011). In gastric cancer, the high expression of USP13 is associated with high invasion, contributing to reduced survival rate of patients. It is supposed that USP13 deubiquitinated and stabilized Snail protein, promoting metastasis in gastric cancer cells (Zhang et al., 2022). Collectively, due to its role in a variety of tumors and neurodegenerative diseases, USP13 has emerged as a potential therapeutic target for diverse tumors.

### Inhibition of USP13

**FIGURE 4:**
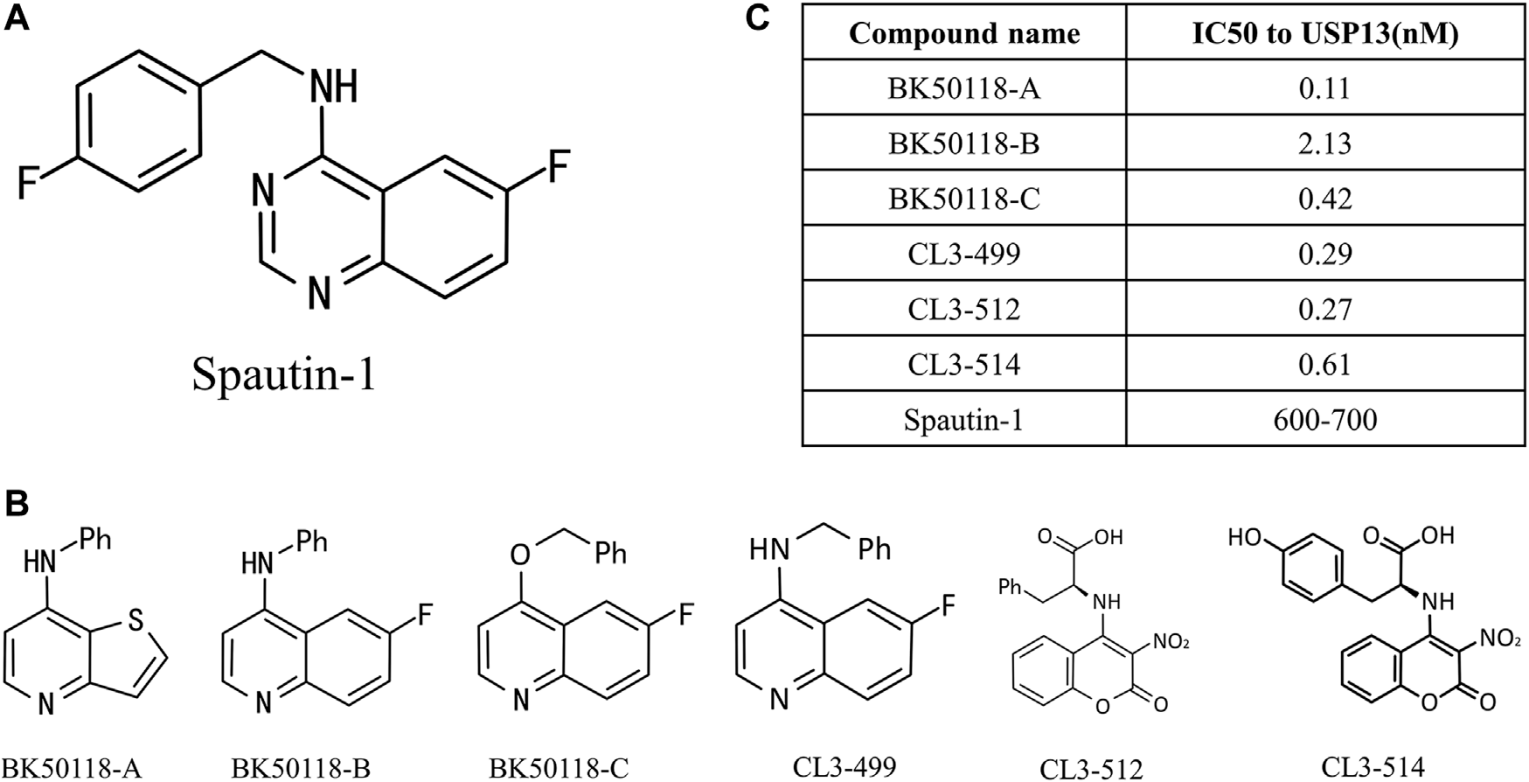
USP13 inhibitors. **(A)** Chemical structure of Spautin-1; **(B)** Spautin-1 derivatives; **(C)** IC50 to USP13 was calculated.

Owing to the significance of USP13 in the above cellular processes and diseases, especially tumors, to seek and develop high potent inhibitors presumably thus offer an attractive strategy for research and treatment of related diseases targeting USP13. Currently, spautin-1 is a widely acknowledged inhibitor of USP13(Liu et al., 2011; Zhang et al., 2018; Guo et al., 2020) (Figure 4A). In 2011, Yuan *et al.* discovered a more efficient autophagy inhibitor, MBCQ, through high-throughput screening. Subsequently, they carried out molecular optimization based on MBCQ and designed many of its derivatives. Among them, C43 is the most superior at selectivity and inhibitory activity, and is named spautin-1 (Liu et al., 2011). Spautin-1 was identified to selectively inhibit the deubiquitination enzymes USP10 and USP13 with an IC50 of 0.6–0.7 μM, and spautin-1 treatment can enhance the ubiquitination-directed degradation of the Beclin1-VSP34 complex and reduce the intracellular concentration of phosphatidylinositol 3-phosphate (PI3P), a crucial component in autophagosome membranes formation (Levine and Kroemer, 2008; Glick et al., 2010; Levy et al., 2017). Remarkably, several studies have successively demonstrated that the use of spautin-1 in combination with chemotherapy can effectively increase tumor cell mortality and attenuate tumor cell migration and xenotransplantation, both in cell models and animal models, suggesting that spautin-1 may be a potential lead compound targeting USP13 (Zhang et al., 2018; Liao et al., 2019; Guo et al., 2020).

More lately, a new study on USP13 inhibitors was reported. Liu *et al.* designed and synthesized six derivatives of spautin-1 (Figure 4B), which exhibit higher inhibition efficiency against USP13 (Figure 4C) and capability of crossing the blood-brain barrier (Liu et al., 2021a), compared to spautin-1, enabling the development of inhibitors in neurodegenerative diseases. They first treated neuroblastoma SH-SY5Y cells with six inhibitors at a concentration ranging from 1 nM to 1 mM, and detected USP13 activity utilizing ELISA assay. The IC50 values of USP13 for these inhibitors ranged from 0.11 to 2.13 nM. Among them, bK50118-C displays the highest inhibitory efficiency against α-synuclein, although its IC50 is not the smallest. Therefore, BK50118-C is selected for the next ADME research. Conclusively, the new USP13 inhibitor BK50118-C designed by Liu *et al.* is the first USP13 inhibitor that can cross the blood-brain barrier, providing a powerful tool for research on USP13-related neurodegenerative diseases in the future.

## Conclusion

Given a decisive role of the ubiquitin–proteasome system (UPS) in protein quality control in eukaryotes, UPS disorder is associated with many diseases, even tumors (Ravid and Hochstrasser, 2008; Harrigan et al., 2018). As a member of this system, the deubiquitinating enzyme USP13 participates in many aspects of cellular processes, as result dysregulation of USP13 gives rise to plenty of diseases through deubiquitination of various critical substrate proteins, including OGDH (Han et al., 2016), ACLY (Han et al., 2016), VPS34 (Liu et al., 2011), TopBP1 (Kim et al., 2021), RAP80 (Li et al., 2017), UBL4A (Liu et al., 2014), and STING (Sun et al., 2017), highlighting that USP13 is emerging as appealing targets for the therapy of the diseases. Consistently, knockdown or pharmacological inhibition of USP13 by spautin-1 retards the growth, differentiation and invasion of many tumors, providing a possibility for antagonizing the drug resistance of tumor cells. Furthermore, recent studies have shown that derivatives of spautin-1 display better USP13 inhibition and the ability to cross the blood-brain barrier, which is presumably beneficial for research on USP13-related neurodegenerative disease (Liu et al., 2021a). However, here a few critical issues are raised. Firstly, since the recombinant expression of USP13 only exhibits weak deubiquitination activity *in vitro*, it should be addressed whether it is in a state of self-inhibition *in vivo* and needs to be activated by other proteins, or its local solubility in the cells requires to be increased for activation. In addition, as neither the structure of the USP13 holoenzyme nor its complex structure with substrate proteins or inhibitors has been determined, it is limited for us to decipher its molecular mechanisms in cell activity. In future, the structure and activity regulation mechanism of USP13 remains to be further elaborated. Moreover, much attention should be paid to the validation utilization of USP13 as a drug target in research on the pathogenesis of diseases, in particular tumors.

We anticipate that this manuscript can supply information on the structure, biology and physiology of USP13, particularly its relation with malignant diseases, paving the way for the clinical transfer of USP13 inhibitors to druggable compounds.

## Glossary

DUB: deubiquitination
USP13: ubiquitin-specific protease 13
ERAD: endoplasmic reticulum-associated degradation
E1: ubiquitin-activating enzyme
E2: ubiquitin-conjugating enzyme
E3: ubiquitin ligase
LUBAC: linear ubiquitin chain assembly complex
USPs: ubiquitin-specific proteases
OTUs: ovarian tumor proteases
UCHs: ubiquitin C-terminal hydrolase
MJDs: Machado–Josephin domain-containing proteases
MINDYs: motifs interacting with the ubiquitin-containing novel DUB family
JAMMs: JAB1, MPN, MOV34 family
ZUP1: zinc finger containing Ub peptidase 1
OGDH: α-ketoglutarate dehydrogenase
ACLY: ATP citrate lyase
VPS34: vacuolar protein sorting 34
TopBP1: topoisomerase IIβ binding protein 1
RAP80: receptor-associated protein 80
Ubl4A: ubiquitin like 4A
MITF: microphthalmia-associated transcription factor
FBXL14: F-box and leucine-rich repeat protein 14
MAPK: mitogen-activated protein kinase
ZnF: Zinc finger
PTEN: phosphatase and tensin homolog deleted on chromosome ten
UBA: ubiquitin-associated
MCL1: myeloid cell leukemia sequence 1
BCL-2: B cell lymphoma 2
NMR: Nuclear Magnetic Resonance
ITC: isothermal titration calorimetry
gp78: glycoprotein 78
Ub-AMC: Ubiquitin-7-amido-4- methylcoumarin
PI3K: Phosphatidylinositol-3-kinase
OVCA: ovarian cancer
CA-USP13: C345A-USP13 mutation
NOD/SCID: nonobese diabetic/severe combined immunodeficiency
NEDD4-1: neural precursor cell expressed developmentally downregulated 4-1
IVDD: intervertebral disc degeneration
DDR: DNA damage repair
BRCA1: breast cancer 1
PARP: Poly (ADP-ribose) polymerase
hHYD: human hyperplastic discs
VCP: valosin-containing protein
Ufd1: ubiquitin fusion degradation 1
Npl4: nuclear protein localization protein 4
Bag6: BCL-2-associated athanogen 6
Ub-Al: ubiquitin aldehyde
AMFR: autocrine motility factor receptor
NSCLC: non-small-cell lung cancer
STING: Stimulator of interferon genes
CLK3: CDC-like kinase 3
TCGA: The Cancer Genomics Atlas
IHC: Immunohistochemical
CAA: cholangiocarcinoma
GSCs: glioma stem cells
HCC: hepatocellular carcinoma
OSCC: oral squamous cell carcinoma
PI3P: phosphatidylinositol 3-phosphate
UPS: ubiquitin–proteasome system.
