# Supplementary figures and images for: USP13: Multiple Functions and Target Inhibition
Source: Front Cell Dev Biol. 2022 Apr 4;10:875124. doi: 10.3389/fcell.2022.875124 (PMC9014248; doi:10.3389/fcell.2022.875124)

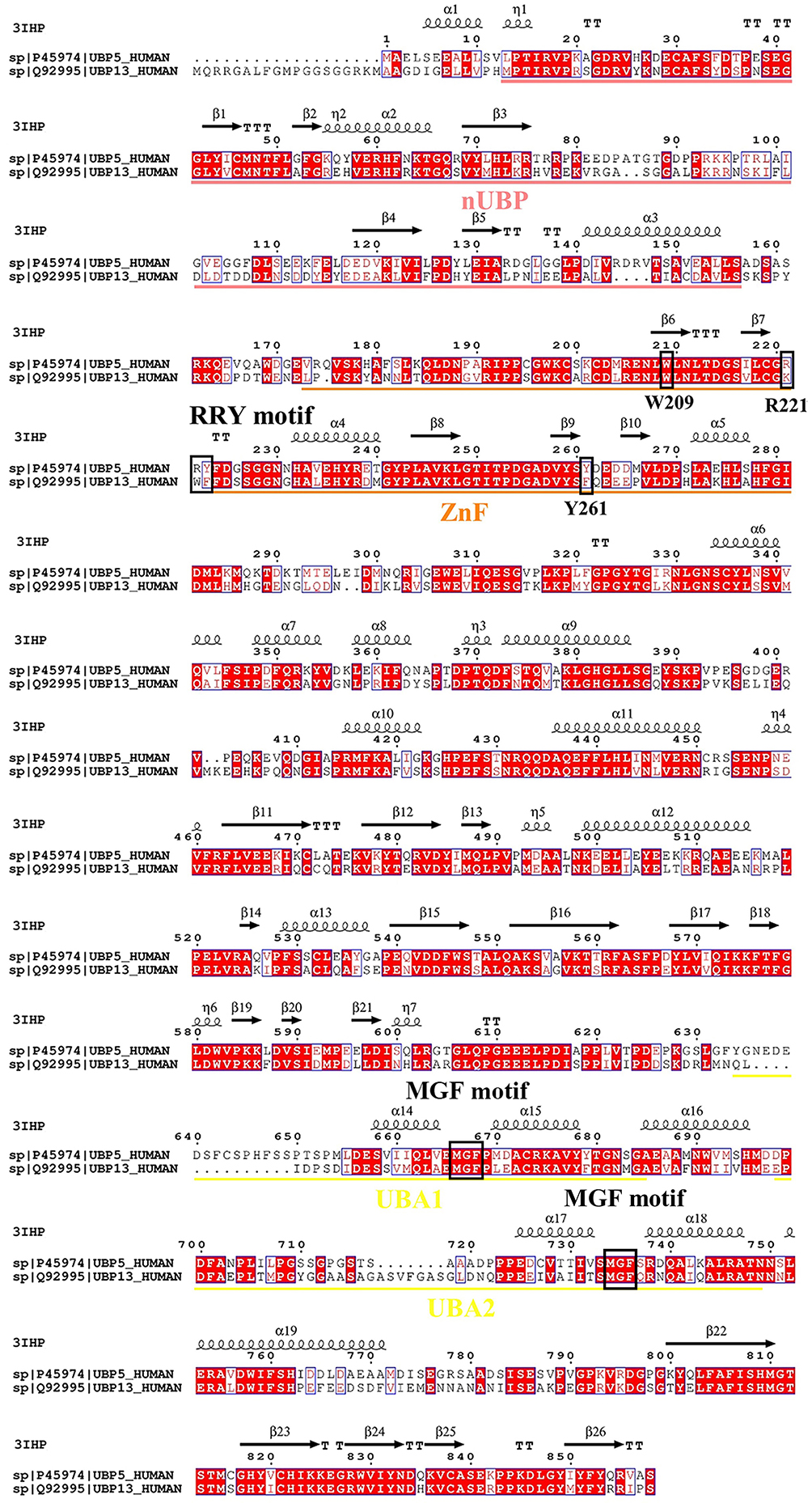

Supplement: Supplementary file 1 [file Image1.jpg]
